# Antimicrobial stewardship approach in reducing incidence of ESBL cases

**DOI:** 10.1017/ash.2025.153

**Published:** 2025-09-03

**Authors:** Lau Hui Shan, Wong Ke Juin, Fong Siew Moy, Sandra Chew Bee Yian

**Affiliations:** 1 Department of PharmacyHospital Wanita dan Kanak kanak, Sabah; 2 Infection control UnitHospital Wanita dan Kanak kanak, Sabah

## Abstract

**Background:** With the increasing cases of multi-resistant organism infection, Antimicrobial Stewardship Programs (ASP) is an important tool in combating this rising challenges. The objective of this study is to review whether the approach of the reduction in usage of second and third generation cephalosporin group antibiotics reduces the incidence of ESBL E.coli and ESBL Klebsiella pneumoniae (KP) cases in Sabah Women and Children Hospital (SWACH) from 2021 until 2023. **Methods:** After discussion with the head of clinical services, the preferred empirical antibiotic of respective unit was switched to ampicilin-sulbactam or amoxicillin clavulanic acid instead of second or third generation cephalosporin. ASP team actively surveillant and monitored the above mentioned antibiotic, cephalosporin usage would be prescribed only to pre-determined indications, while others will be actively switched to ampicilin-sulbactam and amoxicilin- clavulanic acid. This study summarized the usage of Injection second and third generation of cephalosporin group antibiotics from year 2021 to year 2023 and compare with the incidence of ESBL cases per 100 patients admission during the same period. Usage of pediatric antibiotic described in number of vials over total number of admission, while adult antibiotic usage described in Defined daily dose (DDD). Occurrence of ESBL cases described in total number of cases/100 patient admission. The trend will then compared to the baseline ESBL E coli and KP in SWACH in the year prior this intervention. **Results:** For pediatric and adult O&G populations, we observed that the usage of overall cephalosporin group antibiotics showed a decreasing trend since year 2021 till year 2023 and a slight reduction in ESBL E coli and KP was observed at the same period. However, in adult oncology group, despite overall reduction in usage of cephalosporin group antibiotic except injection cefepime, the rate of ESBL E coli showed a slight increase. **Conclusion:** Coordinated efforts between stewardship programs and infection control are essential for reversing conditions that favor the emergence and dissemination of multidrug resistant gram negative bacteria within hospital.

